# Adrenal Insufficiency as a Cause of Acute Liver Failure: A Case Report

**DOI:** 10.1155/2013/487189

**Published:** 2013-02-25

**Authors:** Jamshid Vafaeimanesh, Mohammad Bagherzadeh, Mahmoud Parham

**Affiliations:** Clinical Research Development Center, Qom University of Medical Sciences, Qom 3719764799, Iran

## Abstract

*Introduction*. Many diseases and conditions can contribute to elevated liver enzymes. Common causes include viral and autoimmune hepatitis, fatty liver, and bile duct diseases, but, in uncommon cases like liver involvement in endocrine disorders, liver failure is also seen. Adrenal insufficiency is the rarest endocrine disorder complicating the liver. In the previously reported cases of adrenal insufficiency, mild liver enzymes elevation was seen but we report a case with severe elevated liver enzymes and liver failure due to adrenal insufficiency. Based on our knowledge, this is the first report in this field. *Case Report*. A 39-year-old woman was referred to emergency ward due to drowsiness and severe fatigue. Her laboratory tests revealed prothrombin time: 21 sec, alanine aminotransferase (ALT): 2339 IU/L, aspartate aminotransferase (AST): 2002 IU/L, and ALP: 90 IU/L. No common cause of liver involvement was discovered, and eventually, with diagnosis of adrenal insufficiency and corticosteroid therapy, liver enzymes and function became normal. Finally, the patient was discharged with good general condition. *Conclusion*. With this report, we emphasize adrenal insufficiency (primary or secondary) as a reason of liver involvement in unexplainable cases and recommend that any increase in the liver enzymes, even liver failure, in these patients should be observed.

## 1. Introduction

There is a wide variety of causes for elevated liver enzymes. Approaching a patient with liver dysfunction, the common causes such as viral hepatitis, alcohol abuse, nonalcoholic fatty liver, autoimmune liver diseases, inherited diseases such as alpha-1-antitrypsin deficiency, metabolic liver diseases such as hemochromatosis and Wilson's disease, tumors of the liver or bile ducts, liver damage due to drugs, infection, or cholelithiasis should be considered [1]. Furthermore, in some patients with systemic disease, the liver may involve secondarily. In dealing with unjustified liver dysfunction, systematic diseases affecting liver should be considered, so that, with correct diagnosis along with the primary treatment, liver involvement is cured and inappropriate diagnostic tests and unnecessary treatments are avoided. One of the conditions involving the liver secondarily is endocrine disorders. Hypothyroidism and hyperthyroidism are two known associated endocrine disorders mostly presented in hyperthyroidism. Up to 64% of these patients have ALP elevation and up to 35% have ALT increase. Elevated liver enzymes were reported in hypothyroidism, diabetes mellitus, and Cushing's disease too [1]. One of the endocrine diseases which rarely affects the liver secondarily is adrenal insufficiency [2]. In most reported cases, a slight increase of liver enzymes was observed, and, after treatment of adrenal insufficiency, the liver enzymes abnormalities were resolved. In this paper, we introduce a patient suffering from adrenal insufficiency with sever increase of liver enzymes and liver dysfunction whose liver function normalized after appropriate treatment. According to our information, this is the first case of liver insufficiency and severe increase of liver enzymes due to adrenal insufficiency.

## 2. Case Presentation

The patient is a 39-year-old woman who was referred to the emergency ward of Shahid Beheshti Hospital of Qom due to drowsiness and severe fatigue. Her blood pressure was 134/91 mmHg at the time of admission, and, in her laboratory tests, coagulopathy was detected (Table 1). She was admitted to ICU with coagulopathy and abnormal liver tests diagnosed with liver failure. Necessary tests for evaluation of common reasons of liver insufficiency were performed (Table 2). According to the lab findings, viral, autoimmune, and pharmacological hepatitis and biliary duct diseases were rolled out. The liver sonography was normal. Due to the absence of the common causes of liver failure, she was reexamined and it was revealed that she had been taking Dexamethasone for a long time and discontinued it 3 days prior to admission. So, with suspicion to adrenal insufficiency, some lab tests were requested and the results were ACTH 11 pg/dL (NL = 9–52 pg/dL) and serum cortisol 2.5 *μ*g/dL.

Based on the results, adrenal insufficiency was diagnosed and she underwent prednisolone therapy. After treatment, her liver function tests decreased (Table 1) and, eventually, normalized after 12 days and coagulopathy resolved. She was discharged in good general condition.

## 3. Discussion

Fatty liver is the most common cause of liver involvement presenting with increased liver enzyme because of obesity (nonalcoholic fatty liver) or alcohol consumption. Other causes of liver involvement are viral or autoimmune hepatitis and biliary tract diseases [7]. Approaching a patient with liver dysfunction with unknown cause, the most common causes might be drug-induced liver damage, autoimmune liver disease, and nonalcoholic fatty liver. For example, in a study on 88 patients with abnormal liver function test and unknown cause, the causes were as follows: 34.09% drug-induced liver disease, 22.37% autoimmune liver disease, and 12.5% nonalcoholic fatty liver [8]. One of the less-common reasons of liver involvement is endocrine disorders. Among them, hypothyroidism and hyperthyroidism are the most common reasons of elevated liver enzymes [7]. In addition, Cushing's syndrome can cause nonalcoholic steatohepatitis (NASH) and liver involvement. The increase of liver enzymes was reported in 10–20% of diabetics [1]. O'Beirne et al. suggested that patients with liver failure have a high incidence of adrenal insufficiency, and the degree of adrenal dysfunction is correlated with the severity of the liver disease [9].

Adrenal insufficiency is one of the uncommon reasons of liver involvement [1], but, in patients with liver disease, relative adrenal insufficiency is common in 33% of acute liver failure cases and up to 65% of chronic liver disease and sepsis. Even in the absence of sepsis, relative adrenal insufficiency is common and can affect up to 92% of liver transplant patients (without corticosteroid regimen). This prevalence is so high that some researchers believe that there is a separable pathogenesis in liver patients and propose the term “Hepatoadrenal syndrome” [10]. But liver involvement is not common in patients with adrenal insufficiency and is limited to case reports (Table 3). 

In Olsson et al.'s study in 1990, patients admitted due to slightly increased serum aminotransferases had Addison's disease and the enzymes normalized within 1 week of corticosteroid substitution treatment. They recommended that the possibility of Addison's disease should be considered in patients with obscure slight hypertransaminasemia [3].

Five years later, the second report of liver insufficiency in Addison was published. The researchers introduced 3 patients with abnormal liver enzymes due to adrenal insufficiency, and, after appropriate treatment of adrenal insufficiency, their liver enzymes became normal. Other reports are related to 1998, 2001, and 2006 and the last report was published in 2011 [4–6, 11]. 

All of the introduced patients had mild elevation of liver enzymes, but, in this case, the patient had severe elevation of liver enzymes and liver failure. Based on our information, it is the first case that adrenal insufficiency caused severe elevation of liver enzymes and liver failure. The patient underwent prednisolone therapy, and, after 12 days, her liver enzymes normalized and she was discharged in good general condition.

With this report, we emphasize adrenal insufficiency (primary or secondary) as a reason of liver involvement in unexplainable cases and recommend that any increase in the liver enzymes, even liver failure, in these patients should be observed.

## Figures and Tables

**Table 1:** Evaluation and laboratory data of the patient.

Lab test	Normal range	Admission day	4th day	10th day	12th day (discharge)
PT	12.7–15.4 s	21	15.6	12.6	12.4
INR	—	2.6	1.6	1.1	1
PTT	26.3–39.4 s	33	24	33	28
LDH	115–221 U/L	3003	849	450	440
AST	12–38 U/L	2002	1590	33	30
ALT	7–41 U/L	2339	1081	168	45
Bilirubin total	0.3–1.3 mg/dL	1.3	1.2	0.9	0.9
Bilirubin direct	0.1–0.4 mg/dL	0.5	0.4	0.3	0.2
Sodium	135–148 mmol/L	142	142	140	142
Potassium	3.5–5.3 mmol/L	4	3.9	4.1	4
Calcium total	8.8–10.2 mg/dL	7.7	8.2	8.5	8.4

PT: Prothrombin Time. PTT: Partial Thromboplastin Time. LDH: Lactate dehydrogenase. AST: Aspartate aminotransferase. ALT: Alanine aminotransferase.

**Table 2:** Serology and toxicology results of the patient.

Test	Result	Unit	Reference range
Serum protein electrophoresis			
Albumin	30.9	G/L	36–48
Alpha 1	2.8	G/L	1–3
Alpha 2	8.2	G/L	4–8
Beta	6.8	G/L	5–10
Gamma	11.1	G/L	7–13
Total	59.7	G/L	60–78
Serology			
HAV Ab (I gM)	0.36		Negative: <0.9
HBS Ag	0.4		Negative: <0.9
HCV Ab	Negative		
Hbc Ab	Negative		
Immunofluorescence			
Antinuclear Ab	1/50	Titer	Up to 1/100
Antimitochondrial Ab	1/50	Titer	Negative: <1/100
Antismooth muscle Ab	1/50	Titer	Negative: <1/100
Liver kidney microsomal Ab	1/50	Titer	Negative: <1/100
Drug monitoring			
Phenytoin	Not detected	*μ*g/mL	Toxic level: >20
Phenobarbital	Not detected		Toxic level: >40
Valproate	3.5		Toxic level: >101
Carbamazepine	1.72		Toxic level: >15

**Table 3:** Comparison of published papers about adrenal insufficiency and liver disorders.

Authors	Olsson et al. [3]	Boulton et al. [2]	Castiella et al. [4]	Rizvi and Kirrick [5]	Gurakuqi et al. [6]	Pang et al. [7]	This case
Publication year	1990	1995	1988	2001	2006	2011	2012
Patient characteristics	The first report in this field	The second report in this field	—	Increased liver iron and elevated liver enzymes with adrenal insufficiency	History of 10 years elevated liver enzymes	—	Severe elevated liver enzymes and liver failure associated with adrenal insufficiency
Amount of liver enzyme elevation	Mild	Mild	Mild	Mild	1.5 times more than normal	Mild	Severe
Liver biopsy result	Lymphocyte infiltration at portal region (performed in one patient)	Not performed	Not performed	Lymphocyte infiltration and increased iron deposition	Not performed	Not performed	Not performed
No of patients	4	3	1	1	1	1	1
